# Use of monitoring technology and injury incidence among recreational runners: a cross-sectional study

**DOI:** 10.1186/s13102-021-00347-4

**Published:** 2021-09-28

**Authors:** Richard S. Mayne, Chris M. Bleakley, Mark Matthews

**Affiliations:** 1 School of Health Sciences, University of Ulster at Jordanstown, Newtownabbey, Antrim, BT37 0QB UK; 2 School of Medicine, Dentistry and Biomedical Sciences, Queen’s University Belfast, Belfast, BT9 7BL UK

**Keywords:** Running, Sports training, Monitoring technology, Running-related injury

## Abstract

**Background:**

Monitoring technology is increasingly accessible to recreational runners. Our aim was to examine patterns of technology use in recreational runners, and its potential association with injury.

**Methods:**

We conducted a cross-sectional questionnaire study in a sample of adult runners. Recruitment took place at three different 5 km parkrun event across Northern Ireland. Demographics, technology use, running behaviour and running-related injury (RRI) history were examined. Regression analyses were performed to determine relationships between variables.

**Results:**

Responses were obtained from 192 of 483 eligible finishers (39.8% response rate). Average age was 45.9 years (SD 10.3), with males (47.1 years SD 9.7) slightly older than females (44.8 years SD 10.8). On average, participants ran 3.0 days per week (SD 1.3), with an average weekly distance of 22.6 km (SD 19.7). Males typically ran further (MD 6.2 km/week; 95% CI 0.4 to 12.0) than females. Monitoring technology was used by 87.4% (153/175); with GPS watches the most common device (87.6% (134/153)). Runners using monitoring technology ran further (MD 14.4 km/week; 95% CI 10.3 to 18.5) and more frequently (MD 1.3 days/week; 95% CI 0.7 to 1.9) than those who did not use monitoring technology. There was no significant difference in average age between runners who used monitoring technology and those who did not (MD 4.0 years; 95% CI −0.7 to 8.7). RRI was reported by 40.6% (71/175) of participants in the previous 12 months. In a univariate analysis, none of the selected predictors (age, number of days run per week, distance run per week, or usage of technology to modify training pattern) (*p* > 0.1) were associated with RRI.

**Conclusions:**

This study found a high prevalence of monitoring technology usage among recreational runners. While the incidence of RRI remains high, it is not associated with the usage of monitoring technology. Further prospective research should examine if monitoring technology can reduce RRI incidence among recreational runners in future.

**Supplementary Information:**

The online version contains supplementary material available at 10.1186/s13102-021-00347-4.

## Background

Running is one of the most accessible forms of exercise, with clear physical and mental health benefits [1, 2]. In developed countries, between 12.5 and 25% of the population run recreationally [3]. Participation in recreational running has grown in recent decades [4, 5], evidenced by the current popularity of short 5 km recreational events, like parkrun, as well as longer distance events such as marathons [5–7].

The prevalence of musculoskeletal injury in recreational runners ranges from 19 to 79%, with variance potentially explained by factors such as study duration, sex or running experience [3, 8–11]. A previous systematic review found preliminary evidence that women have a lower risk of RRI than men, but study quality was generally poor, and few studies presented results for men and women separately [11]. Time loss injuries can lead to reduced motivation, depressive symptoms, performance decrements, or potentially, permanent drop out from running, which may impact upon their athletic identity [10, 12, 13]. Identifying important risk factors for running-related injury (RRI) can inform the development of injury prevention interventions for recreational runners, with cumulative population-level health benefits.

A large proportion of runners now run either individually or within small groups, rather than forming part of a larger club [14]. Individual training limits peer-support and guidance from more experienced runners and coaches, increasing the likelihood of inappropriate training behaviours and RRI [1, 9]. However, wearable devices (e.g. watches, phones) incorporating global positioning systems (GPS) and bespoke software [15–20] have been marketed as an alternative to face to face coaching. These technologies are purported to motivate the runner, as well as provide detailed data on performance and tailored advice on modifying future training practices [18]. A potential concern, however, is that overreliance on technology, inaccurate or invalid algorithms, as well as participation in virtual running challenges, also risks poor workload management, overtraining and subsequent injury [19, 20]. This is especially relevant given the recent conflicting theories concerning the relevance of equations such as acute:chronic workload ratios in reducing RRI risk [21]. However, despite the debate regarding the effect of acute and chronic workload on RRI risk, there is an established consensus that risk of RRI increases if load on tissues exceeds their capacity to sustain such load [22].

The primary objective of this cross-sectional study was to examine patterns of use of monitoring technology in a sample of recreational runners participating in parkruns. Secondary aims were to determine whether there is a difference in demographics factors such as sex, running behaviours and incidence of RRI between those who use monitoring technology and those who do not.

## Methods

### Study design

A cross-sectional, paper-based questionnaire study, in a sample of recreational runners participating in parkruns.

### Sampling and recruitment

Recreational runners were recruited at three different parkruns in Northern Ireland, throughout July and August 2019. Parkruns are free, locally organised, timed 5 km running events held every Saturday morning throughout the year. At the time of the study, there were 31 parkrun events in Northern Ireland. We purposively selected three parkruns based on their geographical area, to minimize the risk of crossover between participants. One event was run on a gravel surface, one on a tarmac surface and one on a mixed tarmac/gravel surface. Parkrun participants comprise a wide variety of demographic backgrounds, with children aged older than four, strollers and dogs all allowed [6]. Participants therefore have a wide range of running abilities and levels of experience, with participation encouraged regardless of speed [6]. Participation in the study was voluntary, with no inducements offered. A paper-based questionnaire was designed and piloted for face validity, before being distributed to runners who finished each event. Inclusion criteria were: being aged over 18 years old; having completed the 5 km run; being able to fully complete the questionnaire; having been running regularly for more than six consecutive months. The Ulster University Ethics Filter Committee approved the study and all participants provided informed consent.

### Questionnaire

Individuals showing an interest in participation were provided with an information sheet and given an opportunity to ask questions. All consenting participants were then provided with the questionnaire (paper version) and a pen. The questionnaire (Additional file 1) comprised four sections: demographic details (age, sex); running ability (personal best times in the previous 12 months over a range of distances); running injury history (site of injury, type of advice sought); running habits (frequency, distance per week, use of training plan and/or monitoring technology). The questionnaire was based on previous surveys investigating RRI incidence and use of monitoring technology among recreational runners [15, 16, 18, 20]. An RRI was defined as a sports injury related to running as an independent sport (i.e. not part of a team or field sport e.g. football) occurring within the last 12 months that resulted in cessation of running for more than two weeks [23, 24]. To minimize recall bias, we did not record minor injuries; and participants only reported RRIs resulting in cessation of running for more than two weeks. Previous research has demonstrated negligible cardiovascular deconditioning during the first two weeks of running cessation [25–27]. A researcher remained present throughout, answering questions as required.

### Statistical analysis

Statistical analyses were conducted using SPSS (V.25.0). Baseline characteristics were described using mean (SD) for numerical data and counts (%) for categorical data. The distribution of numerical data was assessed visually using histograms and QQ plots. Independent t-tests were used to examine the relationship between age and running volume, with use of technology. We used logistic regression analysis to explore the relationship between key predictors (age, sex, running volume, use of technology to modify training) and RRI. Predictor variables associated with RRI in univariate analysis (*p* < 0.1), would be included in a multivariate model.

## Results

### Demographic characteristics

Responses were obtained from 192 of 483 eligible parkrun finishers asked to participate in the study (39.8% response rate). Most individuals who declined to participate cited a lack of time. Seventeen responses were excluded, leaving 175 valid responses. The reasons for exclusion were: less than six months of consecutive running experience (n = 10); one or more pages of the questionnaire incomplete (n = 6); illegible response (n = 1). Table 1 summarizes the demographic data, split by sex. Just over half of participants were male (52.6%), with one participant not indicating their sex. The average age was 45.9 years (SD 10.3), with males (47.1 SD 9.7) slightly older than females (44.8 SD 10.8).

**Table 1:** Baseline characteristics

	Total = 175^†^	Male (n = 92)	Female (n = 82)
Age (years)	45.94 (SD 10.3)	47.1 (SD 9.7)	44.8 (SD 10.8)
Number of days running per week	3.0 (SD 1.3)	3.1 (SD 1.5)	2.9 (SD 1.0)
Distance per week (km)	22.6 (SD 19.7)	25.6 (SD 23.4)	19.4 (SD 13.9)
RRI past 12 months yes/no (%)	71/175 (40.6%)	39/92 (42.4%)	31/82 (37.8%)
5 km PB mean^††^ (min:sec)		22:50 (Range 17:30–36:22; Median 21:59; n = 92)	27:06 (Range 18:22–56:00; Median 27:00; n = 82)
10 km PB mean^††^ (hr:min:sec)		0:48:04 (Range 0:36:10– 01:15:00; Median 46:30; n = 65)	0:55:05 (Range 0:38:43–1:16:00; Median 55:00; n = 56)
Half marathon PB mean†† (hr:min:sec)		1:41:45 (Range 1:20:00–2:20:00; Median 01:40:00; n = 33)	2:03:31 (Range 1:26:00–3:05:51; Median 02:00:00; n = 31)
Marathon PB mean^†^^†^ (hr:min:sec)		3:53:22 (Range 2:50:00–5:40:00; Median 03:52:00; n = 22)	04:22:16 Range 3:07:00–6:49:22; Median 03:53:00; n = 19)

^†^One participant did not state their sex

^†^^†^Personal best time in past 12 months

### Running behaviours

Participants ran an average of 3.0 days per week (SD 1.3), with an average weekly distance of 22.6 km (SD 19.7). Although males and females reported a similar number of running days per week (MD 0.2; 95% CI -0.2 to 0.6; *p* = 0.31), males typically ran longer distances (MD 6.2 km/week; 95% CI 0.4 to 12.0, *p* = 0.037), and reported faster personal best times over all distances.

### Technology use

Some form of monitoring technology was used by 87.4% (153/175) of participants. GPS watches were used by 87.6% (134/153). Mobile phone applications were used by 56.2% (86/153), while 12.4% (19/153) used a mobile phone application only. There was lower usage of GPS watches by males (OR 0.80; 95% CI 0.68 to 0.95, *p* = 0.01). Only 3.9% (6/153) of participants were engaged in a physical activity rewards programme, where individuals are given incentives, in the form of financial or non-financial rewards, to be physically active. Runners who used monitoring technology ran further (MD 14.4 km/week; 95% CI 10.3 to 18.5, *p* ≤ 0.001) and more frequently per week (MD 1.3 days/week; 95% CI 0.7 to 1.9, *p* ≤ 0.001), than those who did not use monitoring technology. There was no significant difference in average age between runners who used monitoring technology and those who did not (MD 4.0 years; 95% CI −0.7 to 8.7, *p* = 0.093).

Overall, 43.8% (67/153) of participants who wore monitoring technology used it to modify their training pattern. Runners who used technology to modify their training pattern ran further (MD 13.2 km; 95% CI 7.0 to 19.4 *p* ≤ 0.001) and more frequently each week (MD 0.9 days; 95% CI 0.5 to 1.3, *p* < 0.001) compared to those not using technology to modify training. There was no significant difference in average age between runners who used monitoring technology to modify their training pattern and those who did not (MD 1.4 years; 95% CI −1.8 to 4.5, *p* = 0.389).

### Injury

**Fig. 1:**
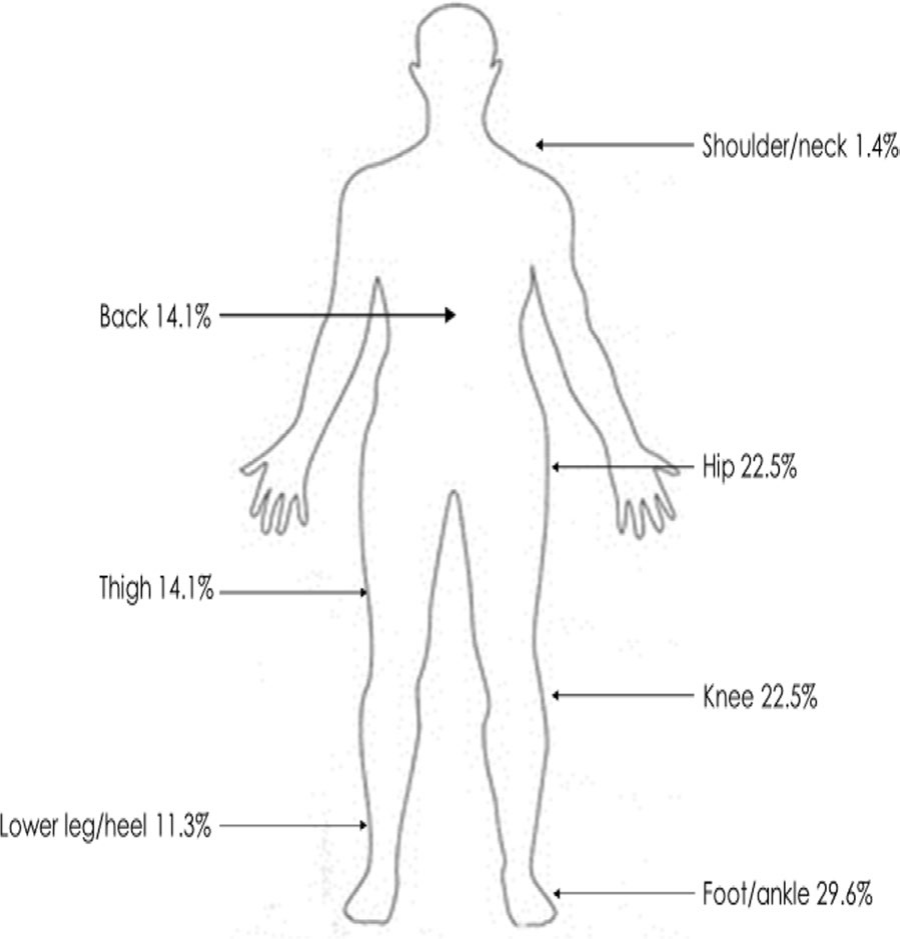
Injury location

Table 1 shows that 40.6% (71/175) of participants reported RRI in the previous 12 months, with a higher proportion of males affected than females (risk difference 4.6%). The foot/ankle was the most injured region (29.6%), followed by hip (22.5%), knee (22.5%), thigh (14.1%), back (14.1%), lower leg/heel (11.3%) and shoulder/neck (1.4%) (Fig. 1). Physiotherapists were the most frequent source of healthcare among runners with RRI (69.0%), followed by medical doctors (29.6%) (Table 2). A significant minority of participants who reported a history of RRI did not seek any healthcare advice to manage their injury (21.1%). Univariate analyses found that none of the selected predictors (age, number of days run per week, distance run per week, or usage of technology to modify training pattern) (*p* > 0.1) were associated with RRI (Table 3), therefore multivariate logistic regression was not undertaken.

**Table 2:** Injury help/advice sought

	n (%)
Physiotherapist	49/71 (69.0)
Doctor	21/71 (29.6)
None	15/71 (21.1)
Online	6/71 (8.5)
Podiatrist	3/71 (4.2)
Chiropractor	3/71 (4.2)
Osteopath	1/71 (1.4)

**Table 3:** Univariate model of potential risk factors for RRI

	Non-injured (n = 104)	Injured (n = 71)	Univariate OR (95% CI)	Univariate *p*-value
Age (years)	45.0 (SD 10.6)	47.4 (SD 9.7)	1.024 (0.99–1.05)	0.13
Number of days running per week	2.9 (SD 1.32)	3.2 (SD 1.32)	1.174 (0.93–1.48)	0.18
Distance run per week (km)	20.9 (SD 17.67)	25.2 (SD 22.2)	1.011 (0.99–1.03)	0.17
Use of technology to modify training pattern (yes/no)	43/61	24/47	0.72 (0.38–1.36)	0.31
Sex† (female/male)	51/53	31/39	0.83 (0.45–1.52)	0.54

^†^1 participant did not state their sex

Values are mean (SD) unless otherwise indicated

## Discussion

This is the first study to evaluate (i) the extent to which parkrun participants use monitoring technology, and (ii) whether there is a difference in demographics, running behaviours and incidence of RRI between those who use technology and those who do not.

### Demographic characteristics

Running, like many other sports, has traditionally had higher rates of participation among males [27]. Findings of this study suggest that participation in running events, particularly over relatively short distances, is increasingly becoming sex-balanced when compared with similar previous studies [16, 28, 20, 29, 30]. The overall average age of 45.9 years compares closely with other similar studies [16, 18, 20]

### Technology use

We found that more recreational runners who participate in parkrun are now using GPS watches (87.6%) compared to previous studies, which varied between 45 and 71% [16, 18, 30, 31]. A similar proportion are using mobile phone applications (56.2%), which previously varied between 19 and 62% [15–17, 30, 31]. Contrary to previous studies [15–18, 20, 30], we found no difference in age between runners who used monitoring technology, and those who did not. This suggests that running technology has moved beyond younger innovators and early adopters and is increasingly accessible to all [32]. This may be due to reduced cost, increased choice, and older members of society becoming increasingly technology literate over time [33]. On average, technology users reported nearly double the weekly running volume compared to non-technology users. This only provides preliminary evidence that running technologies may help to engage and motivate the runner. The cross-sectional nature of our study means that it is difficult to imply causation. For example, users of monitoring technology may have had higher levels of engagement with running prior to the advent of monitoring technology. The differences in reported training volume may also be affected by recall bias, with technology users having regular access to objective data.

### Injury

Despite the higher use of monitoring technology among the participants in this study, the overall RRI incidence of 40.6% aligns with previous studies in recreational runners [9, 10]. This demonstrates that monitoring technology does not currently appear to be correlated with incidence of RRI among recreational runners. Injury locations compare closely with previous studies, with the majority affecting the lower limb [9, 10]. The predictors of RRI identified in previous research, such as age, sex, running experience and training pattern, have often conflicted and contrasted between studies using different methodologies [2, 11, 34–37]. RRI is multifactorial, with the importance of each predictor varying significantly from one runner to the next. The lack of any clear predictors of RRI in our study demonstrates the complex, multifactorial nature of RRI and highlights the difficulties of linking RRI to individual predictor variables. Previous systematic reviews found significant differences in predictors of RRI between different subgroups of runners, depending on running experience, event distance and running surfaces [2, 34]. The population of recreational runners in our study was a heterogenous group, evidenced by a large range in running capabilities, personal best times and weekly training volumes. This heterogeneity of participants, although representative of a typical parkrun, may have reduced our likelihood of identifying individual predictors of RRI. The difference in running surfaces between the different parkrun events in our study (tarmac/gravel etc.) is representative of the varied nature of parkrun events, however this may also have affected the likelihood of identifying individual predictors of RRI.

### Management of injuries

Despite the high rates of usage of monitoring technology, there was a relatively low usage of online resources by individuals who had sustained RRI. The high proportion of injured runners who attended a physiotherapist or doctor indicates that injured runners prefer face-to-face assessment by a registered clinician, and aligns with previous studies examining this area [38–40]. The fact that one in five who sustained RRI did not seek any help or advice indicates a need for further research to explore reasons for this and to identify barriers to seeking help. One potential barrier may be the socioeconomic cost of seeking the advice of a health professional [38–40]. With increasing amounts of cost-free online information relating to running injuries and management, future research could explore its uptake amongst recreational runners, versus more traditional face-to-face methods.

The high uptake of monitoring technology in this study shows that this is an important medium to educate and monitor training practices in recreational runners [31, 41]. However, basic technology tracking univariate factors eg. training distance, or those based around creating personal best competitions with other runners, risk overreaching and overloading beyond internal capacity [19]. Effective reduction of RRI may be more likely with a personalised and evidence-based algorithm that takes into account key external and internal training factors, such as running volume [41, 42].

### Strengths and limitations

To date, few studies have examined the use of monitoring technology by recreational runners. By conducting the survey at parkrun events, participants had a wide range of ages and running abilities, allowing good generalisability to other populations of recreational runners participating in similar events. Using a paper-based questionnaire allowed a broad range of data to be captured, to explore potential relationships between many different variables and ensured there was no bias against individuals who were not familiar with using technology. The overall response rate of 39.8% (192/483) was comparable to previous paper-based questionnaire research [43], however the sample size was relatively small, with a small percentage of the sample not using monitoring technology, which may have resulted in different outcomes compared to non-participants of the study. Self-report of data, such as injury history and training history, may have caused some recall inaccuracy in responses. It has previously been shown that recreational runners can accurately report injury location, but not always injury type [44]. Using a strict definition of RRI (requiring the cessation of running for over two weeks) and the inability of participants to report more than one RRI during the previous 12 months may have resulted in underestimation of RRI. Attempting to explore multiple separate RRIs among the same individual may have given more detail for interpretation, however it also may have led to more inaccuracy in recall.

## Conclusion

Despite the high usage of monitoring technology amongst recreational runners, particularly among female and older runners, there was no significant association with RRI. Monitoring technology, which is becoming increasingly advanced, may play a positive role in reducing incidence of RRI among recreational runners by providing individualised, tailored feedback on running metrics and guiding training plans. Future prospective studies exploring the usage of monitoring technology among recreational runners should assess how this affects their running behaviours and incidence of RRI.

## Supplementary Information

**Additional file 1**. A copy of the questionnaire survey used in the research.

## Data Availability

The anonymised datasets used and/or analysed during the current study are available from the corresponding author on reasonable request.

## Abbreviations

CI: Confidence Intervals
GPS: Global Positioning System
km: Kilometre
MD: Mean Difference
OR: Odds Ratio
PB: Personal Best
RRI: Running Related injury
SD: Standard Deviation

## References

[1] Williams PT (2014). Reduced total and cause-specific mortality from walking and running in diabetes. Med Sci Sports Exerc.

[2] Paluska SA, Schwenk TL (2000). Physical activity and mental health. Sports Med.

[3] Videbaek S, Bueno AM, Nielsen RO, Rasmussen S (2015). Incidence of running-related injuries per 1000 h of running in different types of runners: a systematic review and meta-analysis. Sports Med.

[4] Carter K. Marathons by numbers: running the data. The Guardian [Online], 21 April 2015. https://www.theguardian.com/lifeandstyle/the-running-blog/2015/apr/21/marathons-by-numbers-running-the-data/. Accessed 13 March 2021.

[5] Ingle S. How Parkrun’s 13 became five million and changed weekends for ever. The Guardian [Online], 1 October 2018. https://www.theguardian.com/sport/blog/2018/oct/01/parkrun-five-mlllion-runners/. Accessed 13 March 2021.

[6] Reece LJ, Quirk H, Wellington C, Haake SJ, Wilson F (2019). Bright Spots, physical activity investments that work: Parkrun; a global initiative striving for healthier and happier communities. Br J Sports Med.

[7] Vitti A, Nikolaidis PT, Villiger E, Onywera V, Knechtle B. The “New York City Marathon”: participation and performance trends of 1.2M runners during half-century. Res Sports Med. 2020;28(1):121–137. 10.1080/15438627.2019.1586705 30889965

[8] Buist I, Bredeweg SW, Bessem B, Van Mechelen W, Lemmink KA, Diercks RL (2010). Incidence and risk factors of running-related injuries during preparation for a 4-mile recreational running event. Br J Sports Med.

[9] Francis P, Whatman C, Sheerin K, Hume P, Johnson MI (2019). The Proportion of lower limb running injuries by gender, anatomical location and specific pathology: a systematic review. J Sports Sci Med.

[10] Junior LC, Costa LO, Lopes AD (2013). Previous injuries and some training characteristics predict running-related injuries in recreational runners: a prospective cohort study. J Physiother.

[11] van der Worp MP, ten Haaf DS, van Cingel R, de Wijer A, Nijhuis-van der Sanden MW, Staal JB. Injuries in runners; a systematic review on risk factors and sex differences. PLoS One. 2015;10(2):e0114937. 10.1371/journal.pone.0114937 PMC4338213 25706955

[12] Renton, T., Petersen, B., & Kennedy, S. (2021). Investigating correlates of athletic identity and sport-related injury outcomes: a scoping review. BMJ Open, 11(4), e044199. 10.1136/bmjopen-2020-044199 PMC8043012 33837101

[13] Leddy MH, Lambert MJ, Ogles BM (1994). Psychological consequences of athletic injury among high-level competitors. Res Q Exerc Sport.

[14] Eime RM, Sawyer N, Harvey JT, Casey MM, Westerbeek H, Payne WR (2015). Integrating public health and sport management: Sport participation trends 2001–2010. Sport Manag Rev.

[15] Kemler E, Romeijn K, Vriend I, Huisstede B (2018). The relationship between the use of running applications and running-related injuries. Phys Sportsmed.

[16] Janssen M, Scheerder J, Thibaut E, Brombacher A, Vos S. Who uses running apps and sports watches? Determinants and consumer profiles of event runners’ usage of running-related smartphone applications and sports watches. PloS One. 2017;12(7):e0181167. 10.1371/journal.pone.0181167 PMC5521773 28732074

[17] Wiesner M, Zowalla R, Suleder J, Westers M, Pobiruchin M. Technology adoption, motivational aspects, and privacy concerns of wearables in the german running community: Field Study. JMIR mHealth and uHealth. 2018;6(12):e201. 10.2196/mhealth.9623 PMC6315235 30552085

[18] Dallinga JM, Mennes M, Alpay L, Bijwaard H, De La Faille-Deutekom MB (2015). App use, physical activity and healthy lifestyle: a cross sectional study. BMC Public Health.

[19] Shei RJ (2018). Competitive influences of running applications on training habits. Phys Sportsmed.

[20] Dallinga JM, Van Rijn R, Stubbe J, Deutekom M. Injury incidence and risk factors: a cohort study of 706 8-km or 16-km recreational runners. BMJ Open Sport Exercise Med. 2019;5(1):e000489. 10.1136/bmjsem-2018-000489 PMC6407553 30899549

[21] Wang C, Vargas JT, Stokes T, Steele R, Shrier I (2020). Analyzing activity and injury: lessons learned from the acute: chronic workload ratio. Sports Med.

[22] Cook JL, Docking SI. “Rehabilitation will increase the ‘capacity’ of your ...insert musculoskeletal tissue here....” Defining ‘tissue capacity’: a core concept for clinicians. Br J Sports Med. 2015;49:1484–5. 10.1136/bjsports-2015-094849 26255142

[23] Schmikli SL, Backx FJ, Kemler HJ (2009). National survey on sports injuries in the netherlands: target populations for sports injury prevention programs. Clin J Sport Med.

[24] Kemler E, Blokland D, Backx F, Huisstede B (2018). Differences in injury risk and characteristics of injuries between novice and experienced runners over a 4-year period. Phys Sportsmed.

[25] Grivas GV (2019). The effects of detraining on cardiovascular parameters in distance runners. Med Sci Sports.

[26] Houmard JA, Hortobágyi T, Johns RA, Bruno NJ, Nute CC, Shinebarger MH, Welborn JW (1992). Effect of short-term training cessation on performance measures in distance runners. Int J Sports Med.

[27] Sousa AC, Neiva HP, Izquierdo M, Cadore EL, Alves AR, Marinho DA (2019). Concurrent training and detraining: brief review on the effect of exercise intensities. Int J Sports Med.

[28] Wegner CE, Ridinger LL, Jordan JS, Funk DC (2015). Get serious: gender and constraints to long-distance running. J Leis Res.

[29] Malchrowicz-Mośko E, León-Guereño P, Tapia-Serrano MA, Sánchez-Miguel PA, Waśkiewicz Z. What encourages physically inactive people to start running? An analysis of motivations to participate in Parkrun and City Trail in Poland. Front Public Health. 2020;17(8):581017. 10.3389/fpubh.2020.581017 PMC7707109 33313036

[30] Pobiruchin M, Suleder J, Zowalla R, Wiesner M. Accuracy and adoption of wearable technology used by active citizens: a marathon event field study. JMIR mHealth and uHealth. 2017;5(2):e24. 10.2196/mhealth.6395 PMC5350460 28246070

[31] Clermont CA, Duffett-Leger L, Hettinga BA, Ferber R (2020). Runners’ perspectives on ‘smart’ wearable technology and its use for preventing injury. Int J Hum-Comput Interact.

[32] Ringuet-Riot CJ, Hahn A, James DA (2013). A structured approach for technology innovation in sport. Sports Technology.

[33] Hung LY, Lyons JG, Wu CH (2020). Health information technology use among older adults in the United States, 2009–2018. Curr Med Res Opin.

[34] Kluitenberg B, van Middelkoop M, Diercks R, van der Worp H (2015). What are the differences in injury proportions between different populations of runners? A systematic review and meta-analysis. Sports Med.

[35] Messier SP, Martin DF, Mihalko SL, Ip E, DeVita P, Cannon DW, Love M, Beringer D, Saldana S, Fellin RE, Seay JF (2018). A 2-year prospective cohort study of overuse running injuries: the runners and injury longitudinal study (TRAILS). Am J Sports Med.

[36] Saragiotto BT, Yamato TP, Junior LC, Rainbow MJ, Davis IS, Lopes AD (2014). What are the main risk factors for running-related injuries?. Sports Med.

[37] van Poppel D, van der Worp M, Slabbekoorn A, van den Heuvel SSP, van Middelkoop M, Koes BW, Verhagen AP, Scholten-Peeters GGM (2021). Risk factors for overuse injuries in short- and long-distance running: a systematic review. J Sport Health Sci.

[38] Hespanhol Junior LC, Huisstede BM, Smits DW, Kluitenberg B, van der Worp H, van Middelkoop M, Hartgens F, Verhagen E (2016). The NLstart2run study: economic burden of running-related injuries in novice runners participating in a novice running program. J Sci Med Sport.

[39] Hespanhol Junior LC, van Mechelen W, Postuma E, Verhagen E (2016). Health and economic burden of running-related injuries in runners training for an event: a prospective cohort study. Scand J Med Sci Sports.

[40] Hespanhol Junior LC, Van Mechelen W, Verhagen E (2017). Health and economic burden of running-related injuries in Dutch trailrunners: a prospective cohort study. Sports Med.

[41] Willy RW (2018). Innovations and pitfalls in the use of wearable devices in the prevention and rehabilitation of running related injuries. Phys Ther Sport.

[42] Paquette MR, Napier C, Willy RW, Stellingwerff T (2020). Moving beyond weekly “distance”: optimizing quantification of training load in runners. J Orthop Sports Phys Ther.

[43] Ebert JF, Huibers L, Christensen B, Christensen MB. Paper- or Web-Based Questionnaire Invitations as a Method for Data Collection: Cross-Sectional Comparative Study of Differences in Response Rate, Completeness of Data, and Financial Cost. J Med Internet Res. 2018;20(1):e24. 10.2196/jmir.8353 PMC5801515 29362206

[44] Smits DW, Backx F, Van Der Worp H, Van Middelkoop M, Hartgens F, Verhagen E (2019). Validity of injury self-reports by novice runners: comparison with reports by sports medicine physicians. Res Sports Med.

