# Supplementary material for: Use of monitoring technology and injury incidence among recreational runners: a cross-sectional study
Source: BMC Sports Sci Med Rehabil. 2021 Sep 28;13:116. doi: 10.1186/s13102-021-00347-4 (PMC8480020; doi:10.1186/s13102-021-00347-4)
Supplement: Supplementary file 1 — Additional file 1. A copy of the questionnaire survey used in the research. [file 13102_2021_347_MOESM1_ESM.docx]

**Study title:**

How do recreational runners use technology to inform their training habits and does use of technology affect injury risk?

This questionnaire will take approximately 2-4 minutes to complete. A researcher will provide you with a copy of the questionnaire, a pen and will be present to answer any questions you may have.

1. Your age? (please state) Must be over 18 years old. _____________
2. Gender (please circle)

Male Female

1. Personal best time in the last 12 months (if completed)? (please state)
   1. 5km ____________
   2. 10km ____________
   3. Half-marathon (13.1 miles/21.1km) ____________
   4. Marathon (26.2miles/42.2km) ____________
2. How many consecutive years have you been running regularly (at least once per week)? (please state)

_____________________________________________________

1. Within the last 12 months, have you had a running related injury that caused you to stop running for more than 2 weeks? (please circle)

Yes No

a) If yes, was the injury acute (sudden onset) or chronic (gradual onset)? (please circle)

Acute Chronic

b) If yes, where was the injury? (please circle)

Foot/ankle Lower leg/heel Knee Thigh Hip Back Other (specify) ­­­_____________

c) If yes, did you seek any help/advice for managing your injury? (please circle)

Podiatrist Physiotherapist Doctor Chiropractor Online advice None Other (specify)___________

1. How many days per week do you USUALLY run? (please circle)

1 2 3 4 5 6 7

**PLEASE TURN OVER TO COMPLETE REMAINING QUESTIONS**

1. What is the total distance you USUALLY run per week (approximately)? State whether miles or kilometres.

_____________________________________________________

1. How do you measure the distance you run per week? (please state)

_____________________________________________________

1. Have you used a predefined training plan in the last 12 months (eg. Couch to 5k, Runners World? (please circle)

Yes No

1. Are you engaged in a rewards programme for being physically active (such as Vitality Insurance)? (please circle)

Yes No

1. Do you use a running device, application, or software to track/monitor your running? If so, which one(s)? (please circle/state)
   1. Wrist tracker/watch: ______________________
   2. Phone/app: ______________________
   3. Online/computer software: ______________________
   4. Stopwatch/parkrun times: _______________________
   5. Other: __________________
   6. None
2. If you use technology to monitor your training, have you taken part in any virtual running challenges? (please circle)

None Strava Endomondo Mapmyrun Fitbit Other (specify)_____________

1. If you use technology to monitor your training, do you use the information to modify your training pattern? (please circle)

Yes No

1. If you use technology to monitor your training, please circle your response to the following statement:

Since using technology to monitor my running, I have felt pressurised to run greater distances than I used to run before using technology.

Strongly agree Agree Neither agree nor disagree Disagree Strongly disagree

Thank you for taking time to complete the survey. Have a great day!
